# Systemic Immune-Inflammation Index as a Potential Biomarker for Assessing Disease Activity and Predicting Proteinuria Development in Systemic Lupus Erythematosus

**DOI:** 10.7759/cureus.63401

**Published:** 2024-06-28

**Authors:** Mustafa C Ergun, Eda Aktas, Ahmet T Sahin, Mehmet Sinan İyisoy, Yakup Alsancak, Recep Tunc

**Affiliations:** 1 Department of Rheumatology, Numune State Hospital, Konya, TUR; 2 Department of Internal Medicine, Necmettin Erbakan University, Meram Medical Faculty, Konya, TUR; 3 Department of Cardiology, Beyhekim Training and Research Hospital, Konya, TUR; 4 Department of Medical Education, Necmettin Erbakan University, Meram Medical Faculty, Konya, TUR; 5 Department of Cardiology, Necmettin Erbakan University, Meram Medical Faculty, Konya, TUR; 6 Department of Rheumatology, Necmettin Erbakan University, Meram Medical Faculty, Konya, TUR

**Keywords:** biomarker, disease activity, proteinuria, systemic immune-inflammatory index, systemic lupus erythematosus

## Abstract

Background

Systemic lupus erythematosus (SLE) is a complex autoimmune disease with varied clinical manifestations affecting multiple organ systems. This study aimed to investigate the association between the systemic immune-inflammation index (SII) and disease activity, as well as proteinuria levels in patients with SLE.

Methodology

A total of 141 patients diagnosed with SLE and 99 control subjects were included in this retrospective study. SLE patients were divided into two groups based on the presence (52) or absence (89) of proteinuria. Demographic data, laboratory parameters, and disease activity scores were recorded. SII was calculated based on peripheral blood counts. Statistical analysis was performed to assess the relationship between SII levels and disease activity, as well as proteinuria.

Results

The statistical analysis among the three groups revealed that SII was significantly different in all three groups (p < 0.001). Moreover, within the SLE cohort, patients with proteinuria had significantly higher SII levels compared to those without proteinuria (p = 0.012). Correlation analysis revealed a positive association between SII and both proteinuria and Systemic Lupus Erythematosus Disease Activity Index 2000 (r = 0.215; p = 0.011 and r = 0.186; p = 0.028, respectively). Receiver operating characteristic analysis demonstrated that SII had potential clinical value in diagnosing SLE and predicting proteinuria development.

Conclusions

The findings of this study suggest that SII may serve as a useful biomarker for assessing disease activity and predicting proteinuria development in patients with SLE. Further research is warranted to validate these findings and explore the utility of SII in clinical practice for monitoring disease progression and treatment response in SLE.

## Introduction

Systemic lupus erythematosus (SLE) is an autoimmune disease characterized by a wide range of tissue and organ involvement in the human body. Certain immunological abnormalities, particularly the production of antinuclear antibodies (ANAs), are characteristic features of the disease. Patients present with clinical manifestations ranging from skin involvement to life-threatening kidney, heart, or hematological system involvement. Renal involvement is clinically significant in around half of the individuals diagnosed with SLE and is a primary contributor to both morbidity and mortality in this patient population [1]. Determining the potential development of lupus nephritis beforehand and regulating treatment are crucial for the quality of life and prognosis of patients. Therefore, screening for the development of nephritis is performed at specific intervals in SLE patients.

Systemic Immune-Inflammatory Index (SII) is frequently used as an indicator of mortality in cancer patients [2]. In recent years, the application of SII has been continuously expanding, and several studies have demonstrated its potential utility in predicting the severity of specific diseases and monitoring treatment effects [3-6]. However, SII has not been evaluated in SLE patients with proteinuria in the literature. SII may be useful in evaluating the severity and progression of psoriatic arthritis. Individuals with higher SII levels experience continuous activation of the immune system, leading to chronic inflammation in the tissues [7,8]. This inflammation can cause pain and difficulty in movement by damaging cartilage and bone.

This study aims to investigate the association between SII levels and the diagnosis, disease activity, and proteinuria levels in patients with SLE.

## Materials and methods

Study population

This retrospective, cross-sectional study included a total of 141 patients diagnosed with SLE who presented to the rheumatology outpatient clinic between January 1, 2020, and June 30, 2022, along with 99 individuals in the control group. Patients with other autoimmune rheumatological diseases, lymphoproliferative disorders, malignancies, liver diseases, end-stage kidney disease, diabetic nephropathy, active infections, recent history of blood transfusion, and deficiencies in vitamin B12, folate, and iron were excluded from the study. Patients with laboratory values below the lower limits, including vitamin B12 levels <200 pg/mL, folate levels <4 ng/mL, transferrin saturation <20%, and ferritin <20 mL/ng, were also excluded. The control group was selected from people with similar demographic characteristics and no comorbidities. The study protocol was approved by the Institutional Review Board of Necmettin Erbakan University (protocol number: 2022/3970), and written informed consent was obtained from all participants before their inclusion in the study.

Demographic and clinical data

All patients underwent laboratory evaluations including ANA, anti-dsDNA, complement C3, complement C4, complete urinalysis, 24-hour urine analysis, routine biochemical tests, complete blood count, and vitamin levels (vitamin B12, folate, transferrin saturation, ferritin). ANA was assessed using the indirect immunofluorescence method, while anti-dsDNA was evaluated using the enzyme-linked immunosorbent assay method. Blood samples were collected after an overnight fast. Complete blood count parameters, including neutrophil, lymphocyte, and platelet counts, were measured using an automated hematology analyzer. The SII was calculated using the following formula: SII = (neutrophil count × platelet count)/lymphocyte count. Laboratory results and calculated Systemic Lupus Erythematosus Disease Activity Index 2000 (SLEDAI-2K) disease activity score data of the patients were recorded. SLE diagnosis was established using the 2019 American College of Rheumatology/European League Against Rheumatism criteria. Urinary protein levels were assessed using a 24-hour urine collection. Proteinuria was defined as a 24-hour urinary protein excretion of more than 0.5 g. The presence and degree of proteinuria were monitored and recorded at baseline and during follow-up visits.

Statistical analysis

Data analysis was conducted using SPSS version 22.0 (IBM Corp., Armonk, NY, USA). The normal distribution of variables was assessed using the Kolmogorov-Smirnov test. As all continuous variables did not follow a normal distribution, results were expressed as mean (standard deviation) for continuous variables and as numbers and percentages for categorical variables. The comparison of findings between the two groups was performed using the Wilcoxon rank-sum test, Pearson’s chi-squared test, and Fisher’s exact test. For three-group analysis, one-way analysis of variance and Fisher’s exact test were employed. Spearman correlation analysis test was utilized for correlation analysis. The DeLong test was used for receiver operating characteristic (ROC) analysis, and the Wald test was used for univariable logistic regression analysis. A p-value <0.05 was considered statistically significant.

## Results

Comparison of data between SLE patients and the control group

Among the patients, 133 (94.3%) were female, with a mean age of 36.88 (±11.70) years, and an average proteinuria level of 1,289 (±2,348) mg/day was observed. The demographic data, laboratory results, and disease activity scores of the SLE patients included in the study are presented in Table 1. SLE patients were divided into two groups based on a cut-off value of 500 mg/day for proteinuria obtained from the 24-hour urine test as having or not having proteinuria. Laboratory parameters and disease activity scores were compared between these two groups. Significant differences were observed between the groups in terms of SLEDAI-2K, activation score, ANA titers, anti-dsDNA positivity, and complement C3 levels (p < 0.001, p < 0.001, p = 0.033, p = 0.048, p < 0.001, respectively). Subgroup analysis of SLE patients is presented in Table 2. SLE patients were further divided into two groups based on the presence or absence of proteinuria. Laboratory parameters and SII were compared among SLE patients with proteinuria, those without proteinuria, and the control group. Significant differences were found between the groups in terms of neutrophil, lymphocyte, platelet, sedimentation rate, and SII (p = 0.013, p < 0.001, p = 0.025, p < 0.001, p < 0.001, respectively). The comparison among the three groups is presented in Table 3. Pairwise comparison for SII among the groups revealed statistically significant differences in SII values among all three groups. Statistical data for SII comparison among the groups are presented in Table 4.

**Table 1:** Demographic data and laboratory results of patients with SLE. ^1^: Mean (±SD); ^2^: n (%). ANA = antinuclear antibody; SLE = systemic lupus erythematosus; SLEDAI-2K = Systemic Lupus Erythematosus Disease Activity Index 2000

Parameter	
Age^1^	36.88 (±11.70)
Gender (female)^ 2^	133 (94.3%)
Duration of the illness^1^ (years)	6.33 (±4.71)
SLEDAI-2K^1^	7.38 (±6.33)
Activation score^2^	
No flare	47 (33%)
Mild	61 (43%)
Severe	33 (23%)
Proteinuria^2^	
Absence	89 (63%)
Presence	52 (37%)
Amount of proteinuria^1^	1,289.76 (±2,348.00)
ANA^2^	
<1/160	5 (3.5%)
>1/160	52 (37%)
>1/320	29 (21%)
>1/640	44 (31%)
>1/1,280	10 (7.1%)
Anti-dsDNA^2^	
Negative	47 (33%)
Positive	94 (67%)

**Table 2:** Comparison of SLE patients with and without proteinuria. ^1^: Mean ± SD; n (%); ^2^: Wilcoxon rank-sum test; Pearson’s chi-squared test; Fisher’s exact test. SLE = systemic lupus erythematosus; SLEDAI-2K = Systemic Lupus Erythematosus Disease Activity Index 2000

	Proteinuria absence^1^	Proteinuria presence^1^	P-value^2^
Number of patients	89 (63.1%)	52 (36.9%)	
Duration of the illness (years)	6.31 (±4.93)	6.36 (±4.37)	0.79
SLEDAI-2K	3.55 (±3.29)	13.94 (±4.69)	<0.001
Activation score			
No flare	47 (53%)	0 (0%)	<0.001
Mild	40 (45%)	21(40%)	<0.001
Severe	2 (2.2%)	31(60%)	<0.001
Anti-dsDNA			
Negative	35 (39%)	12 (23%)	0.048
Positive	54 (61%)	40 (77%)	0.048
Complement C3	1.00 (±0.32)	0.75 (±0.38)	<0.001
Complement C4	0.16 (±0.08)	0.16 (±0.15)	0.080

**Table 3:** Comparison of SLE patients with proteinuria, SLE patients without proteinuria, and the control group. ^1^: Mean ± SD; n (%); ^2^: one-way analysis of variance; Fisher’s exact test. WBC = white blood cell; CRP = C-reactive protein; SII = systemic immune-inflammatory index; SLE = systemic lupus erythematosus

	Absence proteinuria^1^	Presence proteinuria^1^	Control group^1^	P-value^2^
Number of patients	52 (21.7%)	89 (37.1%)	99 (41.3%)	
Age	37.19 (±12.70)	38.55 (±14.30)	35.21 (±7.82)	0.15
Gender (female)	47 (90%)	86 (97%)	89 (90%)	0.14
WBC	6.88 (±2.43)	6.68 (±2.95)	6.93 (±1.56)	0.80
Neutrophil	4.94 (±2.24)	4.40 (±2.42)	3.95 (±1.20)	0.013
Lymphocyte	1.41 (±0.77)	1.62 (±0.82)	2.35 (±0.65)	<0.001
Platelet	247.06 (±90.96)	247.38 (±88.03)	275.81 (±63.20)	0.025
Sedimentation	37.87 (±27.55)	18.46 (±15.88)	10.33 (±5.15)	<0.001
CRP	5.76 (±6.08)	5.38 (±9.46)	3.18 (±3.60)	0.091
SII	1,253.44 (±1,356.31)	828.72 (±878.39)	493.62 (±233.10)	<0.001

**Table 4:** Subgroup analysis of SII in SLE patients with proteinuria, SLE patients without proteinuria, and the control group. SLE = systemic lupus erythematosus; SII = systemic immune-inflammatory index; SE = standard error; df = degree of freedom

Contrast	Estimate	SE	df	t ratio	P-value
Proteinuria presence – proteinuria absence	424.720	147.099	235.000	2.887	0.012
Proteinuria presence – control group	759.823	143.725	235.000	5.287	0.000
Proteinuria absence – control group	335.103	123.323	235.000	2.717	0.019

ROC analysis

ROC analysis was conducted to assess the diagnostic accuracy of SII for SLE disease activity and the risk of proteinuria development. For the diagnosis of SLE disease activity, ROC analysis yielded a sensitivity and specificity of 56.8% and 79.8%, respectively, at a cutoff value of 621.6. In the analysis of proteinuria development risk, the sensitivity and specificity of SII at a cutoff value of 1,348.4 were found to be 30.8% and 89.7%, respectively. The results of ROC analyses among the groups are presented in Table 5.

**Table 5:** ROC analysis for the risk of developing SLE and the risk of developing proteinuria in SLE patients. SLE = systemic lupus erythematosus; SII = systemic immune-inflammatory index; AUC = area under the curve; ROC = receiver operating characteristic

	SII optimal cutpoint	Direction	AUC	Sensitivity	Specificity
Diagnosis of SLE	621.679	>=	0.678	0.568	0.798
Development of proteinuria	1,348.421	>=	0.616	0.308	0.897

Logistic regression analysis

Univariable logistic regression analysis was performed for SLE patients with and without proteinuria. The analysis revealed that SII was statistically significant (p < 0.001; odds ratio = 1.00; 95% confidence interval = 1.00-1.00) in terms of the risk of proteinuria. The univariable logistic regression test for proteinuria risk is presented in Table 6.

**Table 6:** Univariable logistic regression analysis conducted for the development of proteinuria. OR = odds ratio; CI = confidence interval; CRP = C-reactive protein; SII = systemic immune-inflammatory index

	OR	95% CI	P-value
Age	0.99	0.97, 1.02	0.57
ANA	1.31	0.95, 1.81	0.10
Anti-dsDNA positive	2.16	1.02, 4.82	0.045
Neutrophil	1.10	0.95, 1.27	0.20
Lymphocyte	0.68	0.40, 1.09	0.11
Platelet	1.00	1.00, 1.00	0.98
Complement C3	0.11	0.03, 0.34	<0.001
Complement C4	0.03	0.00, 2.45	0.12
sedimentation	1.04	1.02, 1.06	<0.001
CRP	1.01	0.96, 1.05	0.80
SII	1.00	1.00, 1.00	0.028

Correlation analysis

Correlation analysis revealed a correlation between SII and proteinuria, as well as between SII and SLEDAI-2K (r = 0.215; p = 0.011 and r = 0.186; p = 0.028, respectively). The results of correlation analysis are presented in Table 7.

**Table 7:** Correlation analysis between SII and other parameters. CRP = C-reactive protein; SII = systemic immune-inflammatory index; SLEDAI-2K = Systemic Lupus Erythematosus Disease Activity Index 2000

	1	2	3	4	5	6
Sedimentation	-					
CRP	r = 0.337 p < 0.000	-				
Complement C3	r =- 0.130 p = 0.131	r = 0.152 p = 0.077	-			
Complement C4	r = -0.087 p = 0.320	r = 0.149 p= 0.087	r = 0.688 p = <0.000	-		
SLEDAI-2K	r = 0.318 p <000	r = 0.152 p = 0.072	r = -0.448 p < 0.000	r = -0.288 p = 0.001	-	
Proteinuria	r = 0.318 p < 0.000	r = 0.137 p = 0.105	r = -0.366 p < 0.000	r = -0.255 p = 0.003	r = 0.709 p < 0.000	-
SII	r = 0.274 p < 0.000	r = 0.084 p = 0.231	r = 0.015 p = 0.861	r = 0.074 p = 0.404	r = 0.186 p = 0.028	r = 0.215 p = 0.011

## Discussion

In our study, SII was found to be significantly higher in SLE patients compared to the control group. Additionally, among SLE patients, the group with proteinuria exhibited significantly higher SII levels compared to the group without proteinuria. Furthermore, a correlation between SII and SLEDAI-2K score was observed in SLE patients.

SII is a relatively novel inflammatory marker in the literature. There have been studies investigating this parameter in various diseases. For instance, a study conducted among 2,642 rheumatoid arthritis (RA) patients and 34,962 control subjects found a correlation between SII and RA, with a calculated cutoff value of 578.25 [9]. Similarly, a study on ankylosing spondylitis patients revealed that SII was elevated in patients compared to the control group and positively correlated with disease activation [10]. Another study on moyamoya patients demonstrated elevated SII levels in both acute and chronic phases of the disease [11]. Similarly, in a study on patients with inflammatory bowel disease, significantly higher SII levels were found [12]. Consistent with these findings, our study also showed increased SII in SLE patients compared to the control group, indicating systemic inflammation associated with this autoimmune disease.

Hematological abnormalities, particularly cytopenias, are commonly observed in SLE patients. All three cell lines can be affected, and cytopenias are often observed. Leukopenia is common in SLE and is frequently associated with disease activity. In SLE patients, lymphopenia can be observed in a wide percentage of cases (20% to 75%) during the active phase of the disease [13]. Thrombocytopenia in SLE can range from mild thrombocytopenia in 25-50% of cases to severe thrombocytopenia in 10% of cases [13,14]. In our study, although the leukocyte count did not show a significant difference between SLE patients and the control group, neutrophil count was increased, while lymphocyte and platelet counts were decreased compared to the control group.

SII is a metric employed for gauging the extent of systemic inflammation in an individual, determined by the platelet count, neutrophil count, and lymphocyte count [15]. In our study, SII was found to be higher in SLE patients compared to the control group, and even higher in patients with proteinuria compared to those without. The positive correlation of SII with SLE in this manner is primarily attributed to the development of lymphopenia in SLE patients. This is because lymphopenia deepens with the development of proteinuria, while the neutrophil and platelet product remains unchanged. In a univariate logistic regression analysis, it was found that certain variables, namely, anti-dsDNA positivity, complement C3 levels, sedimentation rate, and the SII, were significantly associated with the development of proteinuria. Notably, anti-dsDNA positivity, elevated sedimentation rates, and decreased complement C3 levels were identified as factors that increase the likelihood of developing proteinuria.

SLE is an autoimmune disease with systemic involvement, emphasizing the importance of timely diagnosis for initiating treatment [16]. Additionally, determining disease activity is crucial for adjusting treatment intensity, especially considering the significant morbidity and mortality associated with renal involvement in SLE patients. Therefore, there is a constant need for predictive indicators of SLE diagnosis, disease activity, and proteinuria development. Our findings demonstrate a significantly elevated SII in SLE patients compared to the control group. Moreover, a higher SII was observed in patients with proteinuria compared to those without, indicating a positive correlation between SII and proteinuria. The results of the ROC analysis suggest that SII holds specific clinical value in diagnosing SLE and predicting proteinuria development, supporting its utility as a practical parameter in clinical settings. These findings highlight the potential of SII as a research avenue for better predicting disease activity and prognosis in SLE patients.

However, this study has some limitations. First, the study was conducted at a single center and had a retrospective design. This may limit the generalizability of the findings and affect the external validity of the results. Additionally, the sample size in the study was limited, which could affect the statistical power of the findings. Lastly, the fact that the medications used by the patients were not discontinued during the analyses could be a potential confounding factor in determining the results and could affect their interpretation. Considering these limitations, the results of the study should be carefully evaluated.

## Conclusions

This study focuses on examining the SII value in SLE patients. The findings have identified a significantly higher SII in SLE patients compared to the control group. Additionally, a significant difference was observed between SLE patients with and without proteinuria, with a higher SII detected in the group with proteinuria. The results of the correlation analysis indicate a positive relationship between SII and proteinuria, as well as disease activity in SLE patients. ROC analysis demonstrated that SII has a specific clinical applicability value for diagnosing SLE and predicting proteinuria development. Our findings support that SII may serve as a potential inflammatory marker to assess SLE risk and predict proteinuria development in adults.
